# Physical Exercise Alleviates Oxidative Stress and Autonomic Dysregulation in a Rat Model of Inflammatory Bowel Disease

**DOI:** 10.3390/antiox14030328

**Published:** 2025-03-10

**Authors:** Brenda Lois Barros dos Santos, Alda Cássia Alves da Silva, Juliana Soares Severo, Bruno de Sousa Barbosa, Maisa Campêlo de Sousa, Francisco Assis dos Santos Moreira, Lucas Estevão de Sousa, Heron Silva Soares, Antônio Klingem Leite de Freitas, Francisco Leonardo Torres-Leal, Paulo Correia-de-Sá, Armênio Aguiar dos Santos, Moisés Tolentino Bento da Silva

**Affiliations:** 1 Graduate Program in Pharmaceutical Sciences, Federal University of Piaui, Teresina 64049-550, PI, Brazil; brendaloissantos@ufpi.edu.br; 2 Laboratory of Exercise and Gastrointestinal Tract—Department of Physical Education, Federal University of Piaui, Teresina 64049-550, PI, Brazil; aldacassiia.ads@ufpi.edu.br (A.C.A.d.S.); julianasevero@ufpi.edu.br (J.S.S.); estevao.lucas@ufpi.edu.br (L.E.d.S.); heron.soares@ufpi.edu.br (H.S.S.); 3 Graduate Program in Pharmacology, Federal University of Piaui, Teresina 64049-550, PI, Brazil; brunodesousa@ufpi.edu.br (B.d.S.B.); franciscomoreira@ufpi.edu.br (F.A.d.S.M.); torresleal@ufpi.edu.br (F.L.T.-L.); 4 Metabolic Diseases, Exercise and Nutrition Research Group (DOMEN), Laboratory of Metabolic Diseases Glauto Tuquarre, Department of Biophysics and Physiology, Center for Health Sciences, Federal University of Piaui, Teresina 64049-550, PI, Brazil; 5 Department of Physiology and Pharmacology, Federal University of Ceará, Fortaleza 60430-270, CE, Brazil; maisa@ufpi.edu.br (M.C.d.S.); antonioklingem@yahoo.com.br (A.K.L.d.F.); meno@ufc.br (A.A.d.S.); 6 Laboratory of Pharmacology and Neurobiology, (MedInUP/RISE-Health), Department of Immuno-Physiology and Pharmacology, School of Medicine and Biomedical Science—ICBAS, University of Porto, 4050-313 Porto, Portugal; farmacol@icbas.up.pt; 7 Laboratory of Physiology, (MedInUP/RISE-Health), Department of Immuno-Physiology and Pharmacology, School of Medicine and Biomedical Science—ICBAS, University of Porto, 4050-313 Porto, Portugal

**Keywords:** eating behavior, autonomic nervous system, trinitrobenzenesulfonic acid, ileitis, inflammatory bowel diseases, physical exercise

## Abstract

Inflammatory bowel disease (IBD) induces immunological and autonomic imbalances. Exercise is a beneficial strategy for controlling IBD symptoms. We investigated the role of exercise on gastrointestinal (GI) motility changes and autonomic parameters in rats with ileitis. Rats were divided into control, ileitis, and exercise+ileitis groups. Ileitis was induced by TNBS (40 mM, intraileally). The exercise was swimming (1 h/day/4 weeks, 5%/bw). We assessed eating behaviour and oxidative stress. Body composition was assessed by bioimpedance. Autonomic balance and ECG parameters were measured by an electrocardiogram (ECG). Gastrointestinal motility was evaluated using the phenol red technique. In terms of body composition, total body water (TBW), body mass index (BMI), and fat-free mass (FFM) were higher in the ileitis group (216.80 ± 11.44 mL; 24.09 ± 2.15 g/cm^2^; 287.1 ± 14.66 g) (*p* < 0.05) vs. control rats (130.06 ± 28.23 mL; 16.38 ± 2.50 g/cm^2^; 193 ± 42.21 g) and exercise prevented (91.33 ± 12.33 mL; 11.73 ± 0.47 g/cm^2^; 133.8 ± 16.82 g) (*p* < 0.05) these changes. The exercise+ileitis group induces a reduction (*p* < 0.05) in gastric retention vs. ileitis and control (11.22 ± 1.91% vs. 35.17 ± 1.01% and 33.96 ± 1.77%). Ileitis increased intestinal retention in the duodenum (46.3 ± 2.56% vs. 24.98 ± 1.78%) and jejunum (34.22 ± 2.33% and 34.72 ± 2.83% vs. 47.32 ± 1.48%) (*p* < 0.05) and decreased intestinal retention in the ileum (*p* < 0.05) vs. the control group. Exercise+ileitis prevented (*p* < 0.05) changes in the duodenum (24.96 ± 1.66% vs. 46.3 ± 2.56%) and ileum (40.32 ± 3.75% vs. 14.08 ± 0.88%). Ileitis induces high MDA levels (*p* < 0.05) vs. control rats (4.43 ± 0.69 vs. 2.15 ± 0.12 nmol/mg of the tissue). This effect was prevented (*p* < 0.05) in the exercise+ileitis group (2.75 ± 0.21 vs. 4.43 ± 0.69 nmol/mg of the tissue). We observed a reduction in the LF component (*p* < 0.05) in the ileitis group vs. control group (31.32 ± 3.99 vs. 43.43 ± 3.86). The correlation indicated a stronger interrelationship between the autonomic parameter and intestinal retention in the ileum (r: 0.68; *p:* 0.04). The current study suggests intestinal ileitis alters GI motility and autonomic balance, and physical exercise can represent an essential non-pharmacological approach to IBD treatment.

## 1. Introduction

Inflammatory bowel disease (IBD) arises from an inflammatory process within the gastrointestinal tract (GIT), primarily ulcerative colitis (UC) and Crohn’s disease (CD), and is characterized by debilitating and chronic relapsing and remitting inflammation [1,2]. These are multifaceted disorders stemming from various factors, including imbalances in intestinal microbiota, dysregulated immune responses, autonomic dysfunction, appetite dysregulation, and alterations in body composition [3,4].

The intestine communicates with the brain through the autonomic nervous system (ANS)—including the sympathetic and parasympathetic divisions—and the enteric nervous system (ENS) [4]. The sympathetic nervous system (SNS) regulates gastric and immunological functions. In contrast, the parasympathetic nervous system (PNS) modulates anti-inflammatory pathways, gastrointestinal motility, and secretion within the digestive tract through the vagus nerve, which innervates most organs, particularly the gastrointestinal tract [5].

The interaction between the autonomic nervous system and intestinal immunological responses is crucial for maintaining tissue homeostasis and regulating intestinal inflammatory responses. Within the GIT, vagal innervation plays a significant role by directly modulating enteric neurons and indirectly interacting with splenic immune cells, thus maintaining an anti-inflammatory environment [6]. Various strategies, including pharmacological interventions and lifestyle modifications, can be considered to treat intestinal bowel disease.

Scientific evidence suggests that patients with IBDs can have an increased risk of cardiovascular diseases such as myocardial infarction and stroke and overall higher mortality, especially during active disease states [7]. In this context, heart rate variability can be used to investigate changes in the autonomic nervous system in different disease types. Experimental models, such as those of ulcerative colitis and Crohn’s disease, have been used to study the effects of these diseases on sympathetic neurophysiology. In different regions of the GI, such as the intestine, the inflammation has been associated with a decrease in noradrenaline release from sympathetic varicosities [8].

On the other hand, the measurement of heart rate variability (HRV) has been used to assess changes in the sympathovagal balance in rats with colitis by 2,4,6-trinitrobenzene sulfonic acid solution (TNBS) [9]. Moreover, in clinical studies, the results and the use of HRV for the correlation with the changes in intestinal bowel disease are controversial because it is still unclear how HRV is correlated with disease severity within diseased populations or whether changes in HRV are associated with successful treatment or if HRV differs between diseased and healthy states [10].

Changes in body composition have been noted in patients with IBDs, characterized by muscle mass depletion and increased mesenteric adipose tissue [11]. Animal models have investigated this issue to predict disease prognosis [12]. Experimental models of acute and chronic IBD have demonstrated a decrease in intestinal nutrient absorption compared to healthy controls. Furthermore, metabolic recovery during the chronic phase of the disease indicates an adaptive response to inflammation [13]. In addition, alterations in food intake, body composition, and metabolic rate in different stages of the disease can modulate inflammatory properties and the levels of oxidative stress in IBD [14].

Physical exercise has been investigated as a potential intervention to improve intestinal inflammation by its effects on the microbiota, and it has been shown to improve body composition while reducing oxidative stress and inflammation [15]. This is achieved by releasing anti-inflammatory mediators by skeletal muscles, known as myokines, which can exert effects within the GIT [16]. Swimming exercise in animal models leads to neuroendocrine changes. It modifies intestinal motility due to mechanisms involving cholinergic transmission, affecting the resting membrane potential and showing beneficial effects on the gastrointestinal tract [17].

Santos and colleagues [18] also investigated the effects of aerobic physical exercise (swimming without overload for 1 h per day, for five days a week/8 weeks) and strength training (jump 4 × 10 repetitions/5 days a week/8 week with progressive overload) as treatments of animals with ulcerative colitis induced by acetic acid. Despite recent efforts to elucidate the role of physical exercise in the inflammatory process, autonomic regulation, and gastrointestinal functions, there is still a poor understanding of the underlying mechanisms. Therefore, research aimed at elucidating the impact of physical exercise on gastrointestinal and autonomic functions in rats with intestinal ileitis can significantly contribute to the therapeutic management of the disease.

## 2. Materials and Methods

### 2.1. Animals and Ethical Approval

**Figure 1:**
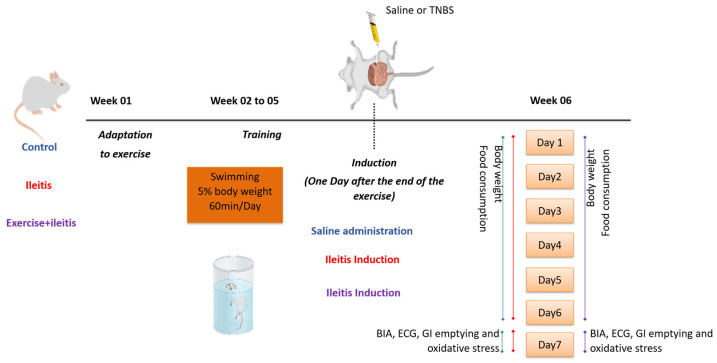
The animals were divided into control, ileitis, and exercise+ileitis groups. The exercise+ileitis group underwent adaptation to the liquid environment for one week, and the following week began the swimming exercise protocol that lasted 4 weeks. The ileitis and ileitis+exercise groups received 2,4,6-trinitrobenzene sulfonic acid (TNBS) in the ileum, and the control group was injected with saline. In week 6 of the protocol, body weight and food consumption were evaluated after ileitis induction. On the 7th day, the animals underwent BIA, ECG, and GI emptying, and biomarkers of oxidative stress were analyzed in the ileal tissue. 2,4,6-trinitrobenzene sulfonic acid (TNBS), bioelectrical impedance (BIA), electrocardiogram (ECG), and evaluation of the gastrointestinal (GI) tract.

Male Wistar rats (*n* = 5–19/group) weighing between 260 and 300 g were utilized in this study. The groups were divided into control, inflammatory ileitis (ileitis), and exercise + inflammatory ileitis (exercise+ileitis). The rats were obtained from the Federal University of Piauí, Brazil. They were housed in collective cages with ad libitum access to water, and food consumption was controlled throughout the study period, both before and after inflammatory ileitis induction. The housing environment was maintained at a controlled temperature of 28 ± 2 °C, with a 12/12 h light/dark cycle. All procedures were conducted according to the recommendations in the “Guide to the Care and Use of Laboratory Animals” and were approved by the Ethics Committee on Animal Use (CEUA) of the Federal University of Piauí (Protocol 687/21). The experimental design is depicted in Figure 1.

### 2.2. Physical Exercise Protocol

The moderate-intensity exercise protocol was based on the methods described by Soares et al. [19]. Before the training, all rats adapted to the liquid medium. In the exercise+ileitis group, the exercise protocol was initiated four weeks before inflammatory ileitis induction. The exercise regimen involved swimming with an overload of 5% of body weight attached to the rats’ tails in collective tanks measuring 100 cm long × 80 cm wide × 80 cm high, with the temperature maintained at approximately 30 ± 2 °C. Each tank accommodated a maximum of 4 rats, with the water depth set at 50 cm. The rats engaged in swimming for 1 h per day, five days per week, and four weeks. Sedentary rats were exposed to shallow water without physical exertion to eliminate any stress-related bias from water contact.

### 2.3. Ileitis Induction Model

**Figure 2:**
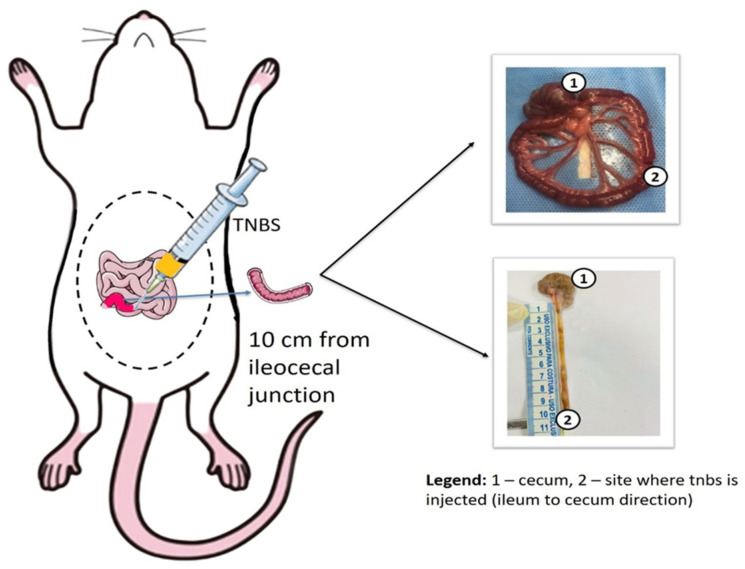
A midline laparotomy was performed, and the terminal ileal loop was exposed. Approximately 10 cm of the ileocolonic junction was exposed for injection of 1 mL of a solution prepared with TNBS into the ileal lumen, and the control animals were injected with 9% saline. The cecum of the animal shown in 1 is located, and 2,4,6-trinitrobenzene sulfonic acid (TNBS) is injected 10 cm in the ileocecal direction shown in 2 for induction of ileitis.

The inflammatory ileitis model was induced by the injection of 2,4,6-trinitrobenzene sulfonic acid (TNBS) directly into the lumen of rats’ ileum, following previously described procedures [20,21], as presented in Figure 2. A detailed description of the inflammatory ileitis model used in the present study is provided by Pontell et al. [22]. After an 8 h fasting period, the rats were anaesthetized with ketamine (80 mg/kg) and xylazine (20 mg/kg, i.m.). A midline laparotomy was then performed to exteriorize the terminal ileal loop, and approximately 10 cm of the ileocolonic junction was exposed. A solution was prepared to consist of (40 µL of the TNBS + 330 µL of ethanol and 730 µL of 0.9% saline for induction of ileitis). The final solution (TNBS 40 µM) was administrated in the ileal lumen using a 27.5 G 1/2 needle. Control rats received 1 mL of 0.9% saline. After administration, the laparotomy was sutured in continuous stitches in the opened muscular layer, with surgical suture and stitch-by-stitch in the external layer. All rats were treated with ampicillin sodium (200 mg/kg, i.m.).

### 2.4. Assessment of Feed Intake Behavior

Feed consumption behavior was assessed seven days after initiating the ileitis induction protocol. The animals were individually placed in cages, and feed intake was monitored daily between 10 and 11 a.m. Each rat had free access to filtered water and 40.0 g of standard pellet feed (Presence Rats and Mice^®^—Agribands Purina do Brazil Ltd.a, Paulínia, SP, Brazil), and the amount consumed during a 24 h interval was quantified [23]. Based on the proximate composition of the feed as 380 kcal/100 g, 63.0 g of carbohydrate/100 g, 23.0 g of protein/100 g, and 4.0 g of fat/100 g of feed, the following nutritional indices were calculated [23]:

1. Energy intake (EI, kcal/day) = average food consumption (g) × dietary metabolizable energy (kcal);
2. Feed efficiency (FE, %) = (average body weight gain (g) × 100)/energy intake (kcal/day);
3. Voluntary food intake (VFI, %) = (average food intake × 100)/average body weight.

### 2.5. Body Composition Assessment

In all groups (control, ileitis, and exercise+ileitis induction, we assessed body composition by the bioimpedance spectroscopy (BIS) method using an ImpediMED^®^ device (Australia/New Zealand), following a protocol previously reported by Santos et al. [24] After the anesthesia with ketamine (80 mg/kg) and xylazine (20 mg/kg/100 g, i.m.), the rats were placed in a supine position on a flat surface for the insertion of four electrodes via hypodermic needles (two in the head and two in the tail). Subsequently, the electrodes were connected to the spectroscope to assess parameters including fat mass (FM; in g), fat-free mass (FFM; in g), total body water (TBW; in mL), extracellular fluid (ECF; in mL), intracellular fluid (ICF; in mL), and body mass index (BMI; in g/cm^2^).

### 2.6. Assessment of Gastrointestinal Motility

The assessment of gastric emptying was conducted seven days after the 4 weeks training period of the control, ileitis, and exercise+ileitis groups. One week after ileitis induction, the groups underwent an 18 h fast. On each experimental day, the rats received a liquid test meal via gavage (1.5 mL of 50 mg/mL phenol red in a 5% glucose solution). After a 10 min postprandial interval, the rats were euthanized with an overdose of thiopental (100 mg/kg, i.p). The gastric retention was evaluated according to the method described by Reynell and Spray [25]. The linear coefficient (α) of the dilution curve defined the concentration of the solution (C = OD) and the amount of phenol red (m) that was recovered from each segment (m = C × volume). The fractional values of gastric or intestinal dye recovery are expressed as follows: dye recovery (%) = 1 − (amount of phenol red recovered in the stomach or intestinal portion/total amount of phenol red recovered from all segments) × 100.

### 2.7. Malondialdehyde (MDA) Assessment

Intestinal tissue samples (ileum) of the control, ileitis, and exercise+ileitis groups were homogenized in a cold 1.15% KCl solution (1 mL per 100 mg of tissue). Briefly, 250 μL of each homogenate was mixed with 1% phosphoric acid (H_3_PO_4_) and 0.6% thiobarbituric acid in aqueous solution. The mixture was stirred and heated in a boiling water bath for 45 min. Subsequently, it was rapidly cooled in an ice-water bath, and 4 mL of n-butanol was added. The mixture was stirred, and the butanol layer was separated by centrifugation at 1200 rpm for 15 min. Optical density was measured at both 535 nm and 520 nm, and the difference between the two readings was recorded as the thiobarbituric acid value. The results were quantified as nanomoles per milligram of tissue (nmol/mg tissue) [26].

### 2.8. Glutathione (GSH) Analysis

The GSH concentration in intestinal tissue samples (ileum) was determined in the three groups using the method described by Olivera et al. [27]. This analysis involves measuring non-protein sulfhydryl groups (NPSH). Samples of 50 to 100 mg of the ileum tissue from the animals were homogenized in 1 mL of 0.02 M EDTA per 100 mg of tissue. Aliquots of 400 μL of the homogenate were mixed with 320 μL of distilled water, and 80 μL of 50% trichloroacetic acid (TCA) was added for protein precipitation. The tubes were centrifuged at 3000 rpm/4 °C for 15 min. Subsequently, 400 μL of the supernatant was combined with 800 μL of 0.4 M Tris buffer (pH 8.9) and 20 μL of dithiol nitrobenzoic acid (DTNB, or Ellman’s reagent). The mixture was stirred for 3 min, and the absorbance was measured at 412 nm using a spectrophotometer. The concentrations of NPSH are expressed as mg NPSH per mg of tissue.

### 2.9. Nitrite/Nitrate (NOx) Evaluation

Nitric oxide production in the intestinal tissue (ileum) of the control, ileitis, and exercise+ileitis groups was assessed indirectly by measuring nitrate (NO_3_^−^) and nitrite (NO_2_^−^) levels, collectively referred to as NOx, using the Griess assay. Serum NO˙ was detected using the modified Griess reaction, where nitrate is reduced to nitrite. This reaction allows the measurement of the total NO˙ (nitrate + nitrite = NOx) present in the tissue analyzed [28]. Tissue samples were macerated in a 0.15 M potassium chloride (KCl) solution, and the resulting homogenate was centrifuged under refrigeration. Subsequently, the supernatant (100 μL) was combined with Griess’ reagent (100 μL) containing phosphoric acid, sulfanilamide, and N-(1-naphthyl) ethylenediamine dihydrochloride. After a 10 min incubation period, the absorbance of the samples was measured at 540 nm. The results are expressed in micromoles of NOx as adapted from [29].

### 2.10. Superoxide Dismutase (SOD) Levels

Intestinal tissue samples (ileum) of the control, ileitis, and exercise+ileitis groups were prepared to prepare a 10% homogenate, then centrifuged at 3000 rpm for 15 min at four °C. Each sample was subsequently mixed with a solution containing phosphate buffer, L-methionine (20 mM), Triton X-100 (1% *v*/*v*), hydroxylamine chloride (10 mM), and EDTA (50 μM). The tubes were incubated in a water bath at 37 °C for 5 min. Riboflavin (50 μM) was added, and all measurements were standardized in a white light box for 10 min. The solution was transferred to an ELISA plate, and a Griess reagent was added. Absorbance readings were taken using an ELISA reader at 550 nm. The SOD unit value (USOD/μg tissue) was then calculated as adapted from [30].

### 2.11. Assessment of the Weight of Organs and Tissues

The organ and tissue weights were measured according to an adaptation of the method described by Santos et al. [24]. After euthanasia, each rat’s heart, duodenum, jejunum, ileum and colon were removed. These tissues were weighed, and the values were adjusted to grams per 100 g of organ or tissue weight.

### 2.12. Cardiac Activity and HRV Assessment

On day six after induction in all groups (control, ileitis, or ileitis+exercise), the rats were anesthetized with ketamine (80 mg/kg, i.m.) and xylazine (20 mg/kg, i.m.) for the bilateral implantation of copper wire coated with insulating material. The wire ends were placed in the thoracic region and right hind paw to monitor electrocardiogram (ECG) activity, with one end sutured to the animal and the other for current capture [31]. One day later, the wires were connected to a transducer and an electrocardiography (ECG) module linked to a signal acquisition system (Powerlab 4/20, ADInstruments^®^, LabChart Pro 8.0) for continuous ECG recordings in lead II derivation. Heart rate (HR; beats per minute, bpm) signals were derived from the R-R″ interval. For the analysis of heart rate variability (HRV), the R-R″ interval was derived from the ECG using fast Fourier transformation (FFT) with the software (LabChart 8.0 ADInstruments^®^, Fortaleza, Brazil). Initially, R waves were detected from a 20 min undisturbed baseline period to create an interval tachogram. The tachogram underwent preprocessing, and the recording was segmented into 1000 data point segments using a ‘Hamming’ tapering window to prevent spectral leakage. The FFT algorithm was then applied to generate periodograms and estimate the R-R″ interval spectrum. ECG monitoring also included a measurement of the PR interval and the correction of values for PR, QRS, QTc, QT intervals, P and JT durations, and amplitudes of Q, R, S, and P, as well as ST height. The Bazett formula was used to determine the QTc interval, as follows:

QTc = QT interval = √(RR interval)

The FFT algorithm generated a spectral density graph depicting frequency components, and the area under the curve was integrated for each oscillatory component, namely the low frequency (LF) and the high frequency (HF). The oscillatory components were then categorized as LF (0.04–0.15 Hz) and HF (0.15–0.4 Hz). Additionally, the data were expressed as the LF/HF ratio (%), reflecting the complex interplay between sympathetic and parasympathetic modulation. The power and relationship of the LF and HF components were expressed in normalized units (nu) and percentages (%). Furthermore, the components included the square root of the mean squares of the differences between adjacent regular RR intervals (RMSSD; ms) and the standard deviation of regular RR intervals (SDRR; ms).

### 2.13. Statistical Analysis

The Kolmogorov–Smirnov test was employed to assess data normality. For comparison between the studied groups, one-way or two-way ANOVA was utilized for variables with normal distribution, followed by the Tukey test for post-hoc analysis. Alternatively, for variables with nonparametric distribution, the one-way or two-way ANOVA tests were followed by the Kruskal–Wallis test. A 95% confidence interval (*p* < 0.05) was considered significant.

## 3. Results

### 3.1. Body Weight and Daily Consumption

**Figure 3:**
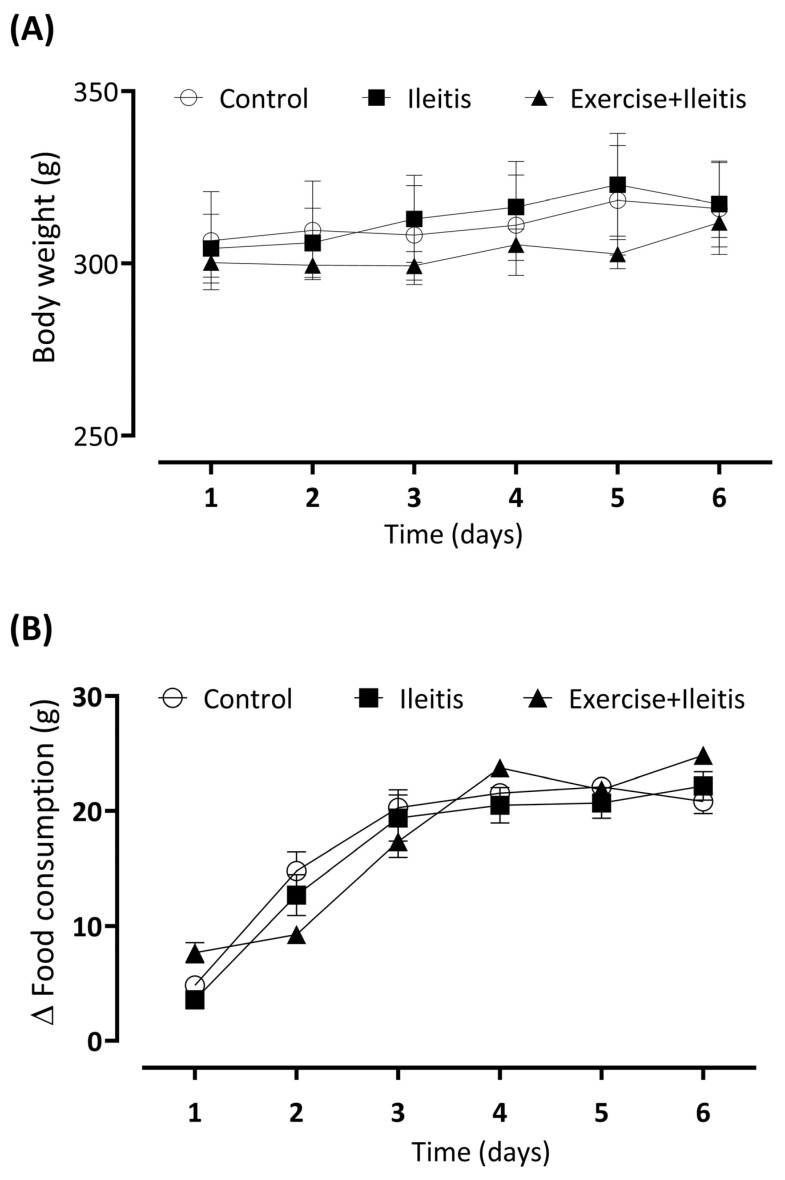
Analysis of body weight and food consumption for 7 days after induction of ileitis in boxes (**A**,**B**). Experimental groups: control (*n* = 13), ileitis (*n* = 18), and exercise+ileitis (*n* = 14). Data are expressed as mean ± (SEM) and were statistically analyzed using the two-way ANOVA test, followed by Tukey’s test. Where values of *p* < 0.05 were considered significant.

Figure 3 presents the results of the variation in body weight and feed intake among the control, ileitis, and exercise+ileitis groups, observed seven days after ileitis induction. We did not observe a difference between body weight and feed intake in all groups during the seven days (Figure 3A,B).

### 3.2. Nutritional Parameters

**Figure 4:**
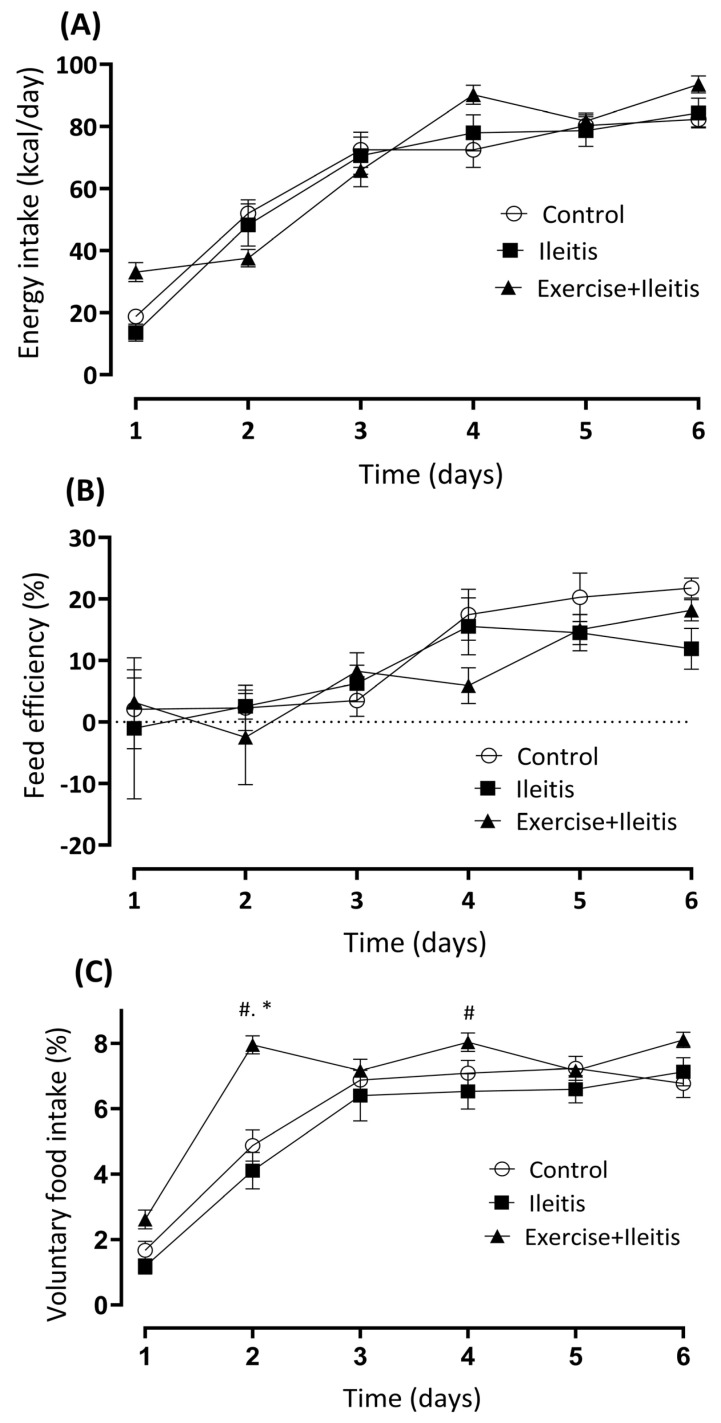
Analysis of energy intake, feed efficiency, and voluntary food intake over 7 days after induction of ileitis (**A**–**C**). Experimental groups: control (*n* = 18), ileitis (*n* = 16), and exercise+Ileitis (*n* = 19). Data are expressed as mean ± (SEM) and were statistically analyzed using the two-way ANOVA test, followed by Tukey’s test. Significance: * vs. control, *p* < 0.05; # vs. ileitis, *p* < 0.05.

Figure 4A,B indicate the energy intake (EI) and feed efficiency. We did not observe differences between all groups six days after induction. Figure 4C depicts a significant high (*p* < 0.05) in VFI on day 2 in the exercise+Ileitis group compared with the ileitis and control groups (7.95 ± 0.28% vs. 4.1 ± 0.6% and 4.8 ± 0.475) and exercise+ileitis vs. ileitis on day 4 (8.03 ± 0.28% vs. 6.5 ± 0.5%).

### 3.3. Body Composition Assessment

Table 1 presents the body composition analysis results for the three groups. Rats in the ileitis group exhibited a significantly high (*p* < 0.05) in total body water (TBW), extracellular fluid (ECF), intracellular fluid (ICF), fat-free mass (FFM), and body mass index (BMI) compared to the control group. Compared to the ileitis group, the exercise+ileitis group prevented this high (*p* < 0.05) in TBW, ECF, ICF, FFM, and BMI. Regarding fat mass (FM), no significant difference was observed between the ileitis and control groups.

### 3.4. Gastrointestinal Parameters

**Figure 5:**
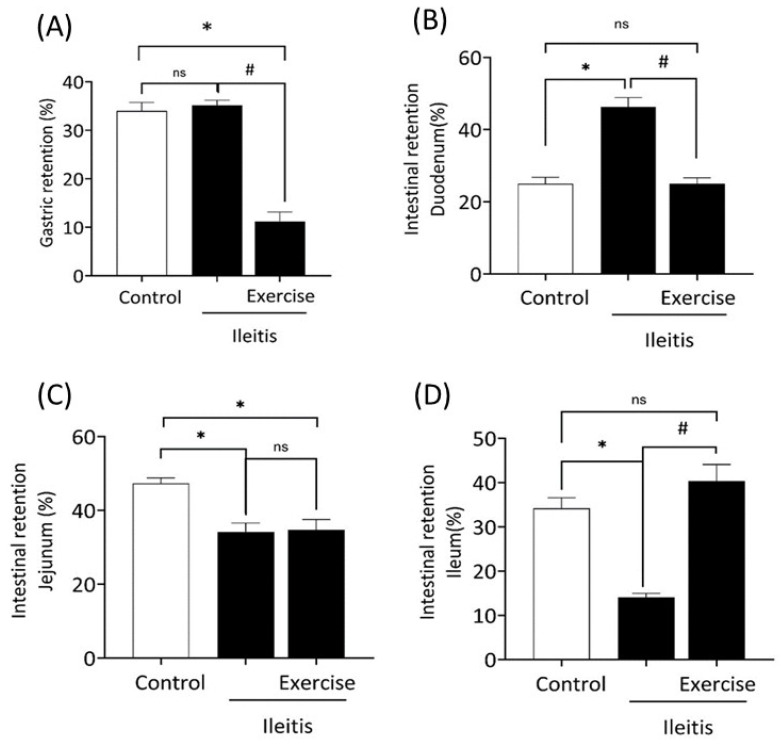
Analysis of gastric retention and intestinal transit in animals with ileitis and previously treated with exercise. Gastric retention (%) (**A**). Intestinal transit in the duodenum portion (**B**). Intestinal transit in the jejunum portion (**C**). Intestinal transit in the ileum portion (**D**). Experimental groups: control (*n* = 12), ileitis (*n* = 10), and exercise+ileitis (*n* = 11). Data are expressed as mean ± (SEM) and were statistically analyzed using the one-way ANOVA test, followed by Tukey’s or Kruskal–Wallis test. * vs. control, *p* < 0.05; # vs. ileitis, *p* < 0.05, ns means not significant.

Figure 5 shows the gastrointestinal parameters, including gastric retention and retention in different intestinal segments, in the control, ileitis, and exercise+ileitis groups. As indicated in Figure 5A, there were no differences in gastric retention between the control and ileitis groups. However, the exercise+ileitis group had lower (*p* < 0.05) gastric retention compared to the ileitis and control groups (11.22 ± 1.91% vs. 35.17 ± 1.01% and 33.9 ± 1.77%). Figure 5B presents the retention in the duodenum. The intestinal retention in the duodenum was significantly higher (*p* < 0.05) in comparison with the control group (46.3 ± 2.56% vs. 24.98 ± 1.78%). The exercise+ileitis group significantly prevented (*p* < 0.05) this effect compared to the ileitis group (24.96 ± 1.66% vs. 46.3 ± 2.56%).

Figure 5C indicates the retention in the jejunum. In both groups, ileitis and exercise+ileitis, we observed a significant reduction (*p* < 0.05) in the jejunal retention compared to the control group (34.22 ± 2.33% and 34.72 ± 2.83% vs. 47.32 ± 1.48%). However, no significant difference was observed in the exercise+ileitis group compared to the ileitis group. Finally, Figure 5D illustrates the intestinal retention in the ileum, for which there was a significant reduction (*p* < 0.05) in ileum retention in the ileitis group in comparison with the control group (14.08 ± 0.88% vs. 34.15 ± 2.39%). Exercise+ileitis prevented this increase (*p* < 0.05) compared with the ileitis group (40.32 ± 3.75% vs. 14.08 ± 0.88%).

### 3.5. Biomarkers of Oxidative Stress

**Figure 6:**
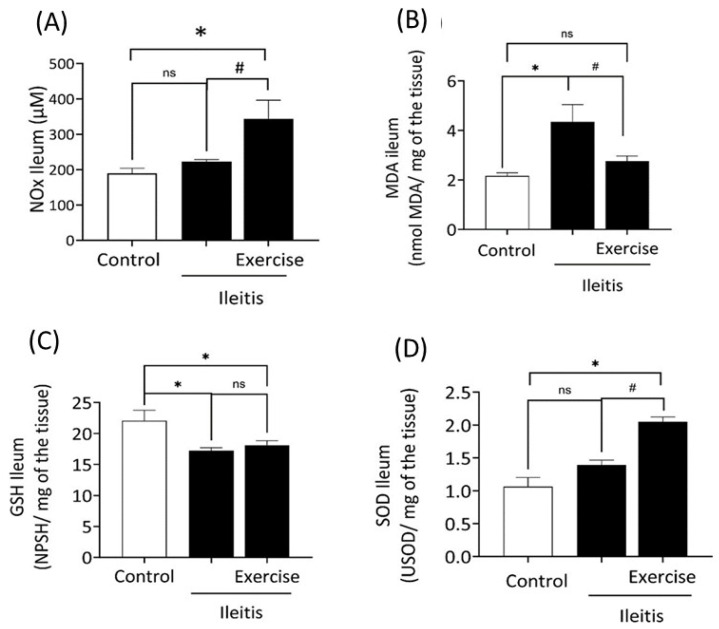
Analysis of biomarkers of oxidative stress in animals’ control, ileitis, and exercise+ileitis on nitrite (μM), of ileal tissue in control (*n* = 9), ileitis (*n* = 7), and exercise+ileitis (*n* =7). (**A**); MDA (nmol/g) of ileal tissue in control (*n* = 7), ileitis (*n* = 6), and exercise+ileitis (*n* =6) (**B**), GSH (NPSH/mg) of ileal tissue in control (*n* = 8), ileitis (*n* = 12), and exercise+ileitis (*n* = 9) (**C**), SOD (U) of ileal tissue in control (*n* = 10), ileitis (*n* = 14), and exercise+ileitis (*n* = 9) (**D**). * vs. control, *p* < 0.05; # vs. ileitis, *p* < 0.05. Data are expressed as mean ± (SEM) and were statistically analyzed using the one-way ANOVA test, followed by Tukey’s or Kruskal–Wallis test. Significance: *p* < 0.05, ns means not significant.

Figure 6A shows NOx activity in the ileum of the control, ileitis, and exercise+ileitis groups. We did not observe a difference between ileitis and the control group.

However, the exercise+ileitis group showed a significant increase (*p* < 0.05) in levels of NOx compared to the ileitis and control groups (342.7 ± 53.35 vs. 222.40 ± 5.86 and 189.60 ± 13.95 µM). Therefore, the exercise+ileitis group showed a significant decrease (*p* < 0.05) in NO production compared to the ileitis and control groups; since NO_3_^−^ and NO_2_^−^ (NOx) are involved in the generation of NO, the higher levels of NOx indicate a lower production of NO. Figure 6B shows the concentrations of malondialdehyde (MDA) in the ileum of the three groups. Compared with the control group, there was a significant high (*p* < 0.05) in MDA concentration in the ileitis group (4.43 ± 0.69 vs. 2.15 ± 0.12 nmol/mg of the tissue). On the other hand, this phenomenon was prevented (*p* < 0.05) in the exercise+ileitis group compared with the values of the ileitis group (2.75 ± 0.21 vs. 4.43 ± 0.69 nmol/mg of the tissue).

Figure 6C shows the concentrations of glutathione (GSH) in the ileum of the three groups. Compared to the control group, there was a significant reduction (*p* < 0.05) in GSH concentration in the ileitis and exercise+ileitis groups (22.06 ± 1.7 vs. 17.26 ± 0.44 and 18.11 ± 0.71 NPSH/mg of the tissue). There was no significant difference between the exercise+ileitis group and the ileitis group. Figure 6D shows the superoxide dismutase (SOD) activity in the ileum of all groups. In the exercise+ileitis group, a significant high (*p* < 0.05) was observed compared to the ileitis and control groups (2.04 ± 0.07 vs 1.39.0 ± 0.07 and 1.06 ± 0.13 USOD/mg of the tissue). We did not observe a difference between the ileitis and control groups.

### 3.6. Weight of the Gastrointestinal Tissue and Heart

Table 2 presents the relative weight of the gastrointestinal tissue and heart in the control, ileitis, and exercise+ileitis groups. In the ileitis group, a significant high (*p* < 0.05) was observed in the ileum and colon compared to the control group. There were not observed differences between the ileitis and the control group in the heart and duodenum. However, in the exercise+ileitis group, a significant high (*p* < 0.05) was observed in the heart, duodenum, and colon compared to ileitis and the control group.

### 3.7. Electrocardiogram (ECG)

Table 3 presents the electrocardiogram (ECG) data of the three groups. No significant differences were observed between the control and ileitis groups concerning the heart rate (HR) and R-R” interval. In the exercise+ileitis group, there was a significant reduction (*p* < 0.05) in HR, and the R-R” interval was significantly high (*p* < 0.05) in comparison with the ileitis group. However, there were no differences between other ECG parameters.

### 3.8. Heart Rate Variability (HRV)

Table 4 reports the heart rate variability (HRV) parameters for the control, ileitis, and exercise+ileitis groups. Notably, the LF (%) and LF (nu) components exhibited a significant reduction (*p* < 0.05) in rats from the ileitis and exercise+ileitis groups compared to the control group. We did not observe differences between all groups in the HF (%) component of the control, ileitis, and exercise+ileitis groups. No differences between groups were observed in the RMSSD and SDRR parameters.

### 3.9. Linear Correlation of Heart Rate Variability and Gastrointestinal Motility

**Figure 7:**
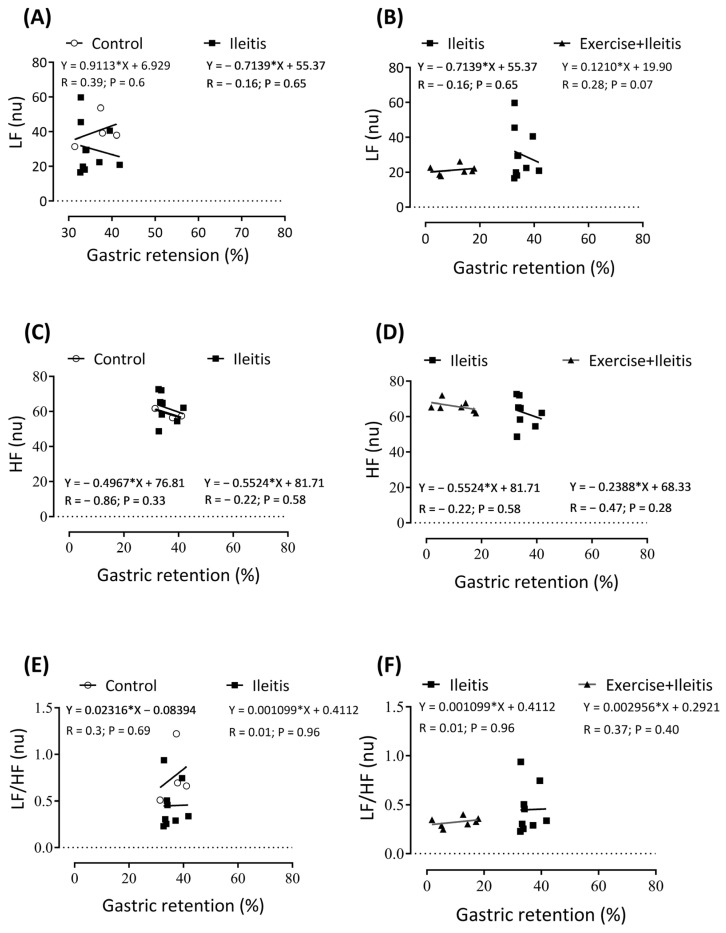
Correlation between gastric retention and variability parameters LF (nu), HF (nu), and LF/HF (%) in rats exercise+ileitis. Control (◯), ileitis (■), and exercise+ileitis (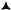). In boxes (**A**,**B**), LF correlation between gastric retention in all groups; in boxes (**C**,**D**) HF correlation between gastric retention in all groups; and in boxes (**E**,**F**) LF/HF correlation between gastric retention in all groups. Each data point indicates one animal. Pearson correlation was used for r and *p* values.

We performed linear regression analysis to compare the relationship between the indirect assessment of HRV components and gastric and intestinal retention results. The results of these components were correlated within the same group. Figure 7 displays the results of linear correlation analysis between gastric retention and the heart rate variability (HRV) parameters LF (nu), HF (nu), and LF/HF (%). No significant correlation was observed between gastric retention and HRV parameters in the three groups.

**Figure 8:**
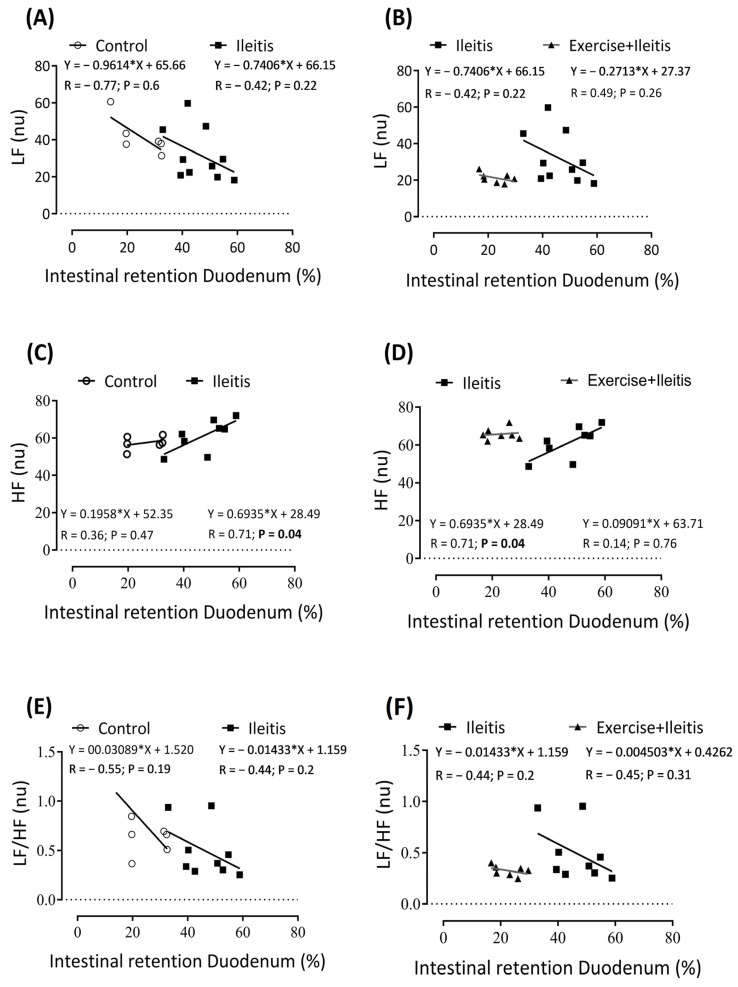
Correlation between intestinal retention duodenum and variability parameters LF (nu), HF (nu), and LF/HF (%) in rats exercise+ileitis. Control (◯), ileitis (■), and exercise+ileitis (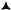). In boxes (**A**,**B**) LF correlation between intestinal retention duodenum in all groups, in boxes (**C**,**D**) HF correlation between intestinal retention duodenum in all groups, and boxes (**E**,**F**) LF/HF correlation between intestinal retention duodenum in all groups. Each data point indicates one individual. Sinificance was found in (**C**,**D**) at *p* < 0.05. Pearson correlation was used for r and *p* values.

Figure 8 illustrates the results of linear regression between intestinal retention in the duodenum and the HRV parameters LF (nu), HF (nu), and LF/HF (%) in the three groups. A positive correlation (*p* < 0.05) was observed (Figure 8C,D) between intestinal duodenum retention and the HF (nu) component in the ileitis group (r: −0.716; *p* = 0.04). However, this correlation was not absent in the control and the exercise+ileitis groups.

**Figure 9:**
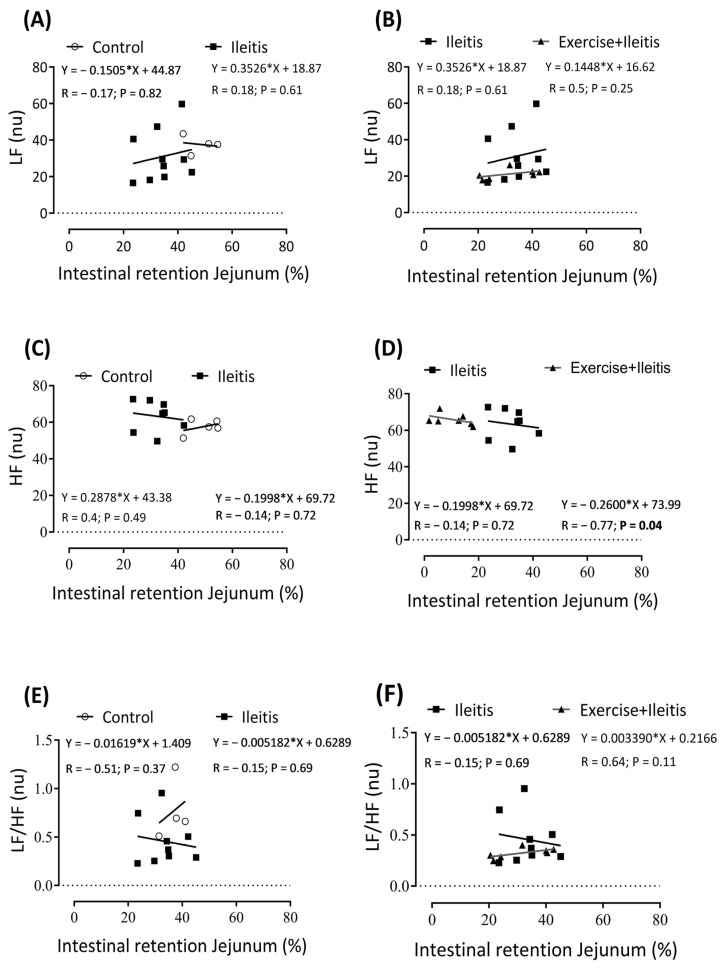
Correlation between intestinal retention jejunum and variability parameters LF (nu), HF (nu), and LF/HF (%) in rats exercise+ileitis. Control (◯), ileitis (■), and exercise+ileitis (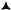). In boxes (**A**,**B**) is the LF correlation between intestinal retention jejunum in all groups, in boxes (**C**,**D**) HF correlation between intestinal retention jejunum in all groups, and in boxes (**E**,**F**) LF/HF correlation between intestinal retention jejunum in all groups. Each data point indicates one individual. Significance was found in (**D**) at *p* < 0.05. Pearson correlation was used for r and *p* values.

Figure 9 depicts the linear correlation results between intestinal retention in the jejunum and heart rate variability (HRV) parameters LF (nu), HF (nu), and LF/HF (%) in the groups. In (Figure 9C), we did not observe changes between intestinal retention in the jejunum and the HF component (nu) in the control and the ileitis group (Figure 9C,D). However, in the exercise+ileitis group (Figure 9D), there was a negative correlation (*p* < 0.05) between jejunum intestinal retention and the HF (nu) component (r: −0.772; *p*: 0.04).

**Figure 10:**
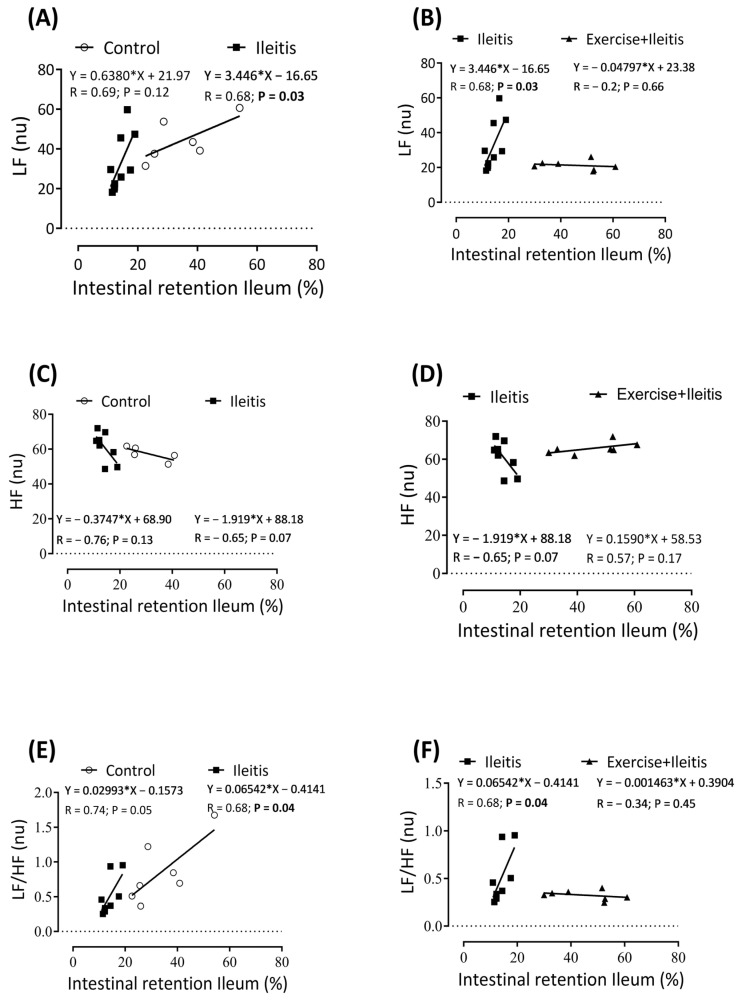
Correlation between intestinal retention ileum and variability parameters LF (nu), HF (nu), and LF/HF (%) in rats exercise+ileitis. Control (◯), ileitis (■), and exercise+ileitis (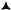). In boxes (**A**,**B**) L, LF correlation between intestinal retention ileum in all groups; in boxes (**C**,**D**), HF correlation between intestinal retention ileum in all groups; and in boxes (**E**,**F**), LF/HF correlation between intestinal retention ileum in all groups. Each data point indicates one individual. Significance was found in (LF) in the ileitis group (**A**,**B)** at *p* < 0.05. Pearson correlation was used for r and *p* values.

Figure 10 illustrates the linear correlation between intestinal retention in the ileum and the heart rate variability (HRV) parameters LF (nu), HF (nu), and LF/HF (%) in the three groups. Figure 10A,B concern the intestinal retention of the ileum and the LF (nu) component. A positive correlation (*p* < 0.05) was observed (r: 0.681; *p*: 0.03). However, this correlation was not absent in the control and the exercise+ileitis group. Furthermore, Figure 10E,F indicate a positive correlation (*p* < 0.05) between the intestinal retention of the ileum and the LF/HF (%) component of the ileitis group (r: 0.68; *p*: 0.04). This correlation was absent between the control group and the exercise+ileitis group.

## 4. Discussion

The present study explored the relationship between physical exercise and gastrointestinal motility in rats with TNBS-induced intestinal ileitis. The jejunum and ileum of the ileitis group showed significantly decreased retention compared to the control rats. The animals submitted to exercise also had a significantly lower jejunum retention rate. However, the retention rate in the ileum was normal in the exercise group. Notably, the retention rate in the duodenum in the ileitis group was significantly higher than the control group, while the retention rate in the duodenum in the exercise group was the same as in the control group.

Our findings showed that the group with intestinal ileitis had no weight variation compared to control rats. This contrasts with the study by Vieira et al. [20], who reported transient weight loss for 3 to 4 days after TNBS, followed by gradual gain. TNBS-induced ileitis in rats does not present a chronic inflammatory phase. It is characterized by a (sub)acute transmural inflammation that is accompanied by functional abnormalities of neuronal activity, with changes in longitudinal muscle contractility that are attributed to the structural thickness of the ileal wall [20,21] that may reflect this transient phase of body weight in these animals, in the proposed model. Although the literature concerning feeding behavior in TNBS-induced ileitis models is scarce, some studies have suggested the impaired absorption of nutrients, water, and electrolytes in IBD [32,33,34,35]. We found that the ileitis group had no differences in energetic intake (EI), feed efficiency (FE), and voluntary feed intake (VFI) compared to the control group. However, the body composition data revealed that these animals had higher total body water (TBW), intracellular fluid (ICF), extracellular fluid (ECF), and body mass index (BMI). In addition to water retention, the animals had a more significant amount of fat-free mass (FFM), which can be explained by the body water content, which is part of this component, together with muscle mass and bone mass [24]. Physical exercise prevented water retention in animals with ileitis, reducing TBW, ICF, and ECF and minimizing TNBS-induced edema.

We did not observe significant differences in gastric retention between the ileitis model and the control group. However, the ileitis group had increased retention in the proximal portion of the intestine. Gastric emptying disorders in IBD usually include delayed emptying [36,37,38], although one study did not find this difference compared to healthy controls [39]. Several hypotheses have been proposed to explain these discrepancies, including suggestions that liquid emptying may be less affected than solid emptying [40], that the disease phases influence gastric emptying characteristics [38], or that the characteristics of the experimental model influence the outcome. Furthermore, changes in ileitis may result from changes in the neural regulation of motility, including autonomic dysfunction [41], as well as hormonal changes in the gastrointestinal tract, such as GLP-1 and ghrelin [38], amplified by the inflammatory processes of the disease [41].

We observed that physical exercise had beneficial effects on the rate of duodenal retention, specifically that it prevented abnormalities in intestinal retention. Discussions in the literature suggest that exercise can induce changes in gastric motility, increasing or decreasing it, with intensity playing a pivotal role in determining these effects. Although not fully understood, the underlying mechanisms probably involve mechanical stimuli [42] and the regulation of autonomic activity [43].

We used the protocol described by Pontell [22] to induce ileitis. In that study, in a model similar to ours, the author observed that at 3 and 7 days after the induction of inflammation by TNBS in the ileum of guinea pigs, the intestinal epithelium microscopically appeared intact, with residues of eosinophilic or neutrophilic cells similar to phagocytosed plasma cells present in the lumen of the epithelial cells. This indicates that epithelial cells play an important role in removing cell debris from the lamina propria of the intestinal mucosa. Since the literature did not report significant microscopic changes in the proposed model [44], we analyzed biomarkers of oxidative stress in inflammation induced by TNBS. We found that MDA concentrations were elevated in the ileum of rats with ileitis, making MDA a pro-oxidant marker. At the same time, GSH levels decreased, making this parameter an antioxidant marker in the model used in the present study. The first line of defense includes antioxidant enzymes such as superoxide dismutase (SOD) and glutathione peroxidase (GPx), which help to prevent the formation of free radicals and neutralize those already formed [45,46]. Exercise induces a reduction in MDA concentrations and a simultaneous increase in SOD activity in the ileum of the exercise+ileitis group. Moderate exercise modulates immune and mitochondrial functions, upregulates the antioxidant system, and has previously been associated with improved oxidative stress in intestinal diseases [18].

IBD is associated with an inflammatory response dependent on NO in the intestinal mucosa, indicating a greater NO-dependent inflammatory response. The regulation of NO production through the expression of inducible nitric oxide synthase (iNOS) represents part of an immediate intestinal antibacterial response. However, NO is also associated with the initiation and maintenance of inflammation in IBD, and iNOS-mediated NO production may occasionally become part of a dysregulated immune response, resulting in increased inflammation [47,48]. In our study, the exercise+ileitis group showed a significant increase in NOx compared to the ileitis and control groups. Since NO_3_^−^ and NO_2_^−^ (NOx) are involved in NO generation, higher NOx levels indicate lower NO production, showing the beneficial effect of physical exercise in ileitis.

Regarding the weight of the organs, many studies have demonstrated that exercise induces hypertrophy and morphological and physiological with a high heart weight [49]. In the current study, exercise induced those effects. Given that our research focused on intestinal and cardiac changes resulting from exercise in the inflammatory ileitis model, the weight of the small intestines and the heart could be anticipated under these conditions. The relative weight of the trained animals’ hearts was greater than that of the other groups, showing that exercise-induced cardiac hypertrophy is an adaptation to physical training. The weight of the ileum in the ileitis group also increased, consistent with fluid retention in the ileitis group. There was a non-significant increase in the weights of the duodenum and colon in the ileitis group, compared to the controls. However, exercise induced a significant increase in weight in the duodenum and colon compared to the ileitis and control groups. The anti-inflammatory effects of training may explain this increase. Swimming in rats with ulcerative colitis improved inflammatory responses in the colon, increasing the length and weight of this organ [18].

Several studies have shown that physical exercise benefits the colon, including preventing constipation and colon cancer [50,51,52,53]. On the other hand, this effect still needs to be evident in intestinal diseases. In the current study, we observed that the exercise+ileitis group showed weight gain compared to the ileitis and control groups. This increase may be due to the volume of feces in the colon, considering that weight values were measured immediately after sacrifice in all groups and were standardized by body weight. Furthermore, the decrease in sympathetic activity in the exercise+ileitis group could exacerbate the parasympathetic component, which would justify the greater motility of the intestinal contents to the colon, causing it to have greater weight.

Regarding the autonomic nervous system assessment, through an indirect evaluation of the autonomic components and heart rate (HR), we did not detect significant changes in rats with TNBS-induced ileitis. However, in the heart rate variability (HRV) parameters, another study reported a reduction in the LF (%) and LF (nu) components, as well as in the LF/HF balance (%), suggesting the existence of autonomic nervous system (ANS) dysfunction in animals with intestinal ileitis [54]. In the study of Ciesielczyk [10], these authors showed that animals with colitis caused by TNBS presented significant increases in LF (nu) and LF/HF compared to controls, suggesting a change in sympathovagal balance induced for the disease. In the current study, we noted a reduction in these components without changes in the HF component.

To correlate the responses of the ANS components with the adverse effects in the GIT, we conducted a correlation between the LF (nu), HF (nu), and LF/HF (nu) components in comparison with gastrointestinal motility analysis in all groups separately. This analysis revealed positive correlations between the LF (nu) component and the intestinal retention rate in the ileum for the same group with ileitis, as well as between the LF/HF ratio (nu) and the intestinal retention rate in the ileum in the presence of ileitis. These findings support our understanding of ANS dysfunction, showing increased sympathetic activity in the ileum represented by the LF (nu) component and that in the exercise+ileitis group, this effect is prevented (Figure 10E,F).

The literature on the genesis and implication of ANS dysfunction is still controversial, especially in the balance between sympathetic and parasympathetic activity in IBD [55,56,57,58,59]. However, this dysfunction affects gastrointestinal motility and patient symptoms [60]. The impact of physical exercise on HRV has been extensively studied, showing variation according to the type and intensity of training [61]. Research has focused on how IBD alters the ANS due to inflammatory processes and their impact on the intestine [62,63].

Moderate exercise training is currently considered the primary adjunct in improving autonomic function and has considerable effects on HRV, including increasing vagal tone and modulating sympathovagal balance activity. Furthermore, exercise training has anti-inflammatory effects [64], indicating a possible mechanism by which exercise improves autonomic function [65].

Our results demonstrate that physical exercise at moderate-intensity aerobic helps attenuate the effects of ileitis on body composition, primarily by improving fluid retention, as observed in animals with ileitis. Ileitis affects gastrointestinal motility, reduces sympathetic tone, and contributes to autonomic dysfunction.

Despite being a well-established model with characteristics resembling CD in humans, the inflammatory ileitis induction model still requires further studies to understand the nuances and limitations of experimental models. In the current study, we had a limitation of the difficulty in establishing a long-term model where the effect of exercise as a form of treatment and not just as prevention could be evaluated. Furthermore, the results related to the autonomic nervous system are indirect measurements based on cardiac parameters. The measurement of direct sympathetic and parasympathetic activity in the gastrointestinal tract is a study limitation. In addition, there is a lack of studies in this experimental model associated with physical exercise and the possible physical and molecular adaptations that training causes in IBD, but which provide us with essential insights for management in humans.

## 5. Conclusions

In conclusion, our findings highlight the role of moderate-intensity aerobic physical exercise in mitigating the impact of ileitis on body composition, particularly by improving fluid retention in affected animals and modulating gastrointestinal motility, sympathetic tone, and autonomic dysfunction. However, further studies are needed to elucidate the underlying mechanisms involved in these effects.

## Figures and Tables

**Table 1:** Body composition parameters.

Parameters	Control	Ileitis	Exercise+Ileitis
TBW (mL)	130.60 ± 28.23	216.80 ± 11.44 *	91.33 ± 12.33 ^#^
ECF (mL)	52.19 ± 15.75	96.32 ± 5.16 *	38.99 ± 7.49 ^#^
ICF (mL)	78.37 ± 12.56	120.50 ± 6.60 *	52.34 ± 5.58 ^#^
FFM (g)	193.00 ± 42.21	287.10 ± 14.66 *	133.80 ± 16.82 ^#^
FM (g)	172.40 ± 7.61	152.80 ± 11.82	174.90 ± 15.35
BMI (g/cm^2^)	16.38 ± 2.50	24.09 ± 2.15 *	11.73 ± 0.47 ^#^

Total water body (TWB); extracellular fluid (ECF); intracellular fluid (ICF); fat-free mass (FFM); fat mass (FM); body mass index (BMI). Experimental groups: control (*n* = 7), ileitis (*n* = 8), and exercise+ileitis (*n* = 5). Data are expressed as mean ± (SEM) and were statistically analyzed using the one-way ANOVA test, followed by Tukey’s test. Significance * vs. control, *p* < 0.05; ^#^ vs. ileitis, *p* < 0.05.

**Table 2:** Relative weight of organs per 100 g of animal weight.

Tissue	Control	Ileitis	Exercise+Ileitis
Heart (g/100 g)	0.3530 ± 0.022	0.3453 ± 0.02	0.6176 ± 0.084 *^#^
Duodenum (g/100 g)	0.223 ± 0.0177	0.256 ± 0.03	0.4855 ± 0.031 *^#^
Ileum (g/100 g)	0.3687 ± 0.024	0.5034 ± 0.037 *	0.3563 ± 0.046 ^#^
Colon (g/100 g)	0.513 ± 0.034	0.574 ± 0.026	1.385 ± 0.118 *^#^

Heart (g/100 g), duodenum (g/100 g), ileum (g/100 g), and colon (g/100 g). Experimental groups: control (*n* = 10), ileitis (*n* = 10), and exercise+ileitis (*n* = 10). Data are expressed as mean ± (SEM) and were statistically analyzed using the one-way ANOVA test, followed by Tukey’s. Significance * vs. control, *p* < 0.05; ^#^ vs. ileitis, *p* < 0.05.

**Table 3:** Electrocardiogram parameters.

Parameters	Control	Ileitis	Exercise+Ileitis
HR (bpm)	391.1 ± 10.06	368.6 ± 8.315	342 ± 5.235 *^#^
R-R” Interval (s)	0.154 ± 0.003	0.164 ± 0.003	0.176 ± 0.002 *^#^
PR Interval (s)	0.049 ± 0.001	0.047 ± 0.001	0.044 ± 0.001 *
Duration P (s)	0.015 ± 0.001	0.013 ± 0.001	0.011 ± 0.001 *
QRS Interval (s)	0.016 ± 0.001	0.016 ± 0.001	0.0161 ± 0.001
QTc Interval (s)	0.057 ± 0.002	0.053 ± 0.001	0.052 ± 0.001
QT Interval (s)	0.021 ± 0.001	0.021 ± 0.001	0.022 ± 0.001
ST height (mV)	0.001 ± 0.006	0.003 ± 0.002	0.01 ± 0.003

Heart rate—HR (bpm). R-R” interval (s). PR Interval (s). Duration P (s). QRS interval (s). QTc interval (s). QT interval (s). ST height. Experimental groups: control (*n* = 8), ileitis (*n* = 12), and exercise+ileitis (*n* = 19). Data are expressed as mean ± (SEM) and were statistically analyzed using the one-way ANOVA test, followed by Tukey’s. Significance * vs. control, *p* < 0.05; ^#^ vs. ileitis, *p* < 0.05.

**Table 4:** Heart rate variability parameters.

Parameters	Control	Ileitis	Exercise+Ileitis
VLF (%)	39.32 ± 8.0	47.07 ± 7.45	52.14 ± 5.90
LF (%)	24.44 ± 2.07	14.42 ± 1.59 *	11.53 ± 1.19 *
LF (nu)	43.43 ± 3.86	31.32 ± 3.99 *	19.85 ± 0.82 ^#,^*
HF (%)	37.70 ± 4.35	33.54 ± 5.52	30.75 ± 4.12
HF (nu)	57.41 ± 1.49	61.75 ± 2.77	67.38 ± 1.26
LF/HF (%)	0.70 ± 0.10	0.48 ± 0.08	0.34 ± 0.03 *
RMSSD (ms)	4.57 ± 0.61	4.49 ± 0.71	6.32 ± 0.92
SDRR (ms)	8.27 ± 1.10	6.50 ± 0.78	8.30 ± 0.83

Low-frequency component—LF (%). LF (nu). High-frequency component—HF (%). HF (nu). The ratio between low and high frequency is LF/HF (%). The square root of the mean square of the differences between adjacent normal RR intervals—RMSSD (ms). The standard deviation of normal RR intervals—SDRR (ms). Experimental groups: control (*n* = 8), ileitis (*n* = 11), and exercise+ileitis (*n* = 16). Data are expressed as mean ± (SEM) and were statistically analyzed using the one-way ANOVA test, followed by Tukey’s. Significance * vs. control, *p* < 0.05; ^#^ vs. ileitis, *p* < 0.05.

## Data Availability

The raw data supporting the conclusions of this article will be made available by the authors upon request.

## Abbreviations

The following abbreviations are used in this manuscript:

IBD	Inflammatory bowel disease
GI	Gastrointestinal
TNBS	2,4,6-trinitrobenzene sulfonic acid
ECG	Electrocardiogram
TBW	Total body water
BMI	Body mass index
FFM	Fat-free mass
GIT	Gastrointestinal tract
UC	Ulcerative colitis
CD	Crohn’s disease
HPA	Hypothalamic–pituitary–adrenal
ANS	Autonomic nervous system
ENS	Enteric nervous system
SNS	Sympathetic nervous system
PNS	Parasympathetic nervous system
HRV	Heart rate variability
CEUA	Ethics Committee on Animal Use
EI	Energy intake
FE	Feed efficiency
VFI	Voluntary food intake
BIS	Bioimpedance spectroscopy
FM	Fat mass
ECF	Extracellular fluid
ICF	Intracellular fluid
MDA	Malondialdehyde
GSH	Glutathione
NPSH	Non-protein sulfhydryl
TCA	Trichloroacetic acid
DTNB	Dithiol-nitrobenzoic acid
NOx	Nitrite/nitrate
KCl	Potassium chloride
SOD	Superoxide dismutase
FFT	Fourier transformation
LF	Low frequency
HF	High frequency
RMSSD	Square root of the mean squares of the differences between adjacent regular RR intervals
SDRR	Standard deviation of regular RR intervals
GPx	Glutathione peroxidase
HR	Heart rate
MDPI	Multidisciplinary Digital Publishing Institute
DOAJ	Directory of open access journals
TLA	Three letter acronym
LD	Linear dichroism
